# Methylphenidate improves prefrontal cortical cognitive function through α2 adrenoceptor and dopamine D1 receptor actions: Relevance to therapeutic effects in Attention Deficit Hyperactivity Disorder

**DOI:** 10.1186/1744-9081-1-2

**Published:** 2005-04-22

**Authors:** Amy FT Arnsten, Anne G Dudley

**Affiliations:** 1 Department of Neurobiology, Yale Medical School, New Haven, CT 06510, USA

## Abstract

**Background:**

Methylphenidate (MPH) is the classic treatment for Attention Deficit Hyperactivity Disorder (ADHD), yet the mechanisms underlying its therapeutic actions remain unclear. Recent studies have identified an oral, MPH dose regimen which when given to rats produces drug plasma levels similar to those measured in humans. The current study examined the effects of these low, orally-administered doses of MPH in rats performing a delayed alternation task dependent on prefrontal cortex (PFC), a brain region that is dysfunctional in ADHD, and is highly sensitive to levels of catecholamines. The receptor mechanisms underlying the enhancing effects of MPH were explored by challenging the MPH response with the noradrenergic α2 adrenoceptor antagonist, idazoxan, and the dopamine D1 antagonist, SCH23390.

**Results:**

MPH produced an inverted U dose response whereby moderate doses (1.0–2.0 mg/kg, p.o.) significantly improved delayed alternation performance, while higher doses (2.0–3.0 mg/kg, p.o.) produced perseverative errors in many animals. The enhancing effects of MPH were blocked by co-administration of either the α2 adrenoceptor antagonist, idazoxan, or the dopamine D1 antagonist, SCH23390, in doses that had no effect on their own.

**Conclusion:**

The administration of low, oral doses of MPH to rats has effects on PFC cognitive function similar to those seen in humans and patients with ADHD. The rat can thus be used as a model for examination of neural mechanisms underlying the therapeutic effects of MPH on executive functions in humans. The efficacy of idazoxan and SCH23390 in reversing the beneficial effects of MPH indicate that both noradrenergic α2 adrenoceptor and dopamine D1 receptor stimulation contribute to cognitive-enhancing effects of MPH.

## Background

Methylphenidate (MPH) is a leading treatment for Attention Deficit Hyperactivity Disorder (ADHD). Although this compound has been used for decades, the neural mechanisms underlying MPH's therapeutic actions are still unknown. Recent advances in our understanding of the neurobiology of ADHD, and the identification of appropriate MPH doses for use in rodents, now allow the examination of therapeutic actions in animals.

Converging evidence has demonstrated that ADHD symptoms arise from dysregulation of prefrontal cortical (PFC)/striatal and cerebellar circuits (reviewed in [1]. The PFC uses working memory to guide behavior and attention, inhibiting inappropriate responses and sustaining attention over long delays, particularly under conditions of interference from distractors [2,3]. Deficits in PFC function lead to poor impulse control, distractibility, hyperactivity, forgetfulness and poor organization and planning [4]. There is general agreement that ADHD involves weakened PFC function e.g. [5], and speculation that medications might strengthen PFC abilities. Imaging studies have shown that MPH produces more efficient PFC function in both ADHD patients [6] and control subjects [7], consistent with this view.

Many researchers have assumed that MPH acts by blocking dopamine (DA) transporters (reviewed in [8]). Indeed, elegant PET imaging studies of DA transporter occupancy in striatum have shown that MPH acts at this site [9]. However, the striatum contains very few noradrenergic (NE) transporters, and thus the important actions of MPH on the NE system have received far less attention. At the present time, imaging studies are unable to reliably visualize the low levels of NE and DA actions in cortex, although there is the suggestion that there may be fewer catecholamine terminals in the PFC of adults with ADHD [10]. Thus, animal studies are of particular importance for understanding MPH actions in PFC.

Recent animal studies by Kuzcenski and Segal [11] have identified the low, oral doses of MPH which 1) produce plasma levels in rats similar to those observed in children taking MPH, and 2) decrease locomotor activity in rats just as they do in humans. Oral administration was key, as MPH administration by injection produces much higher blood and brain MPH levels [12]. Prior to appreciation of this research, MPH doses in rat studies were generally too high, and were usually administered by injection, producing kinetics and drug levels relevant to drug abuse but not to ADHD e.g. [13-15]. These injected, effectively higher doses produce locomotor hyperactivity with stimulant treatment e.g. [16,17], and it has been assumed that there were species differences that would impede research. Thus, the identification of the appropriate dose regimen for MPH treatment in rats opens a new field of research that may more quickly elucidate MPH therapeutic mechanisms.

Although previous research focused on MPH amplification of DA actions, more recent biochemical studies using low doses of MPH show more potent effects on hippocampal NE than on striatal DA [18], while increasing both DA and NE release in the PFC ([19] and C.W. Berridge, personal communication). Both NE and DA have a critical influence on PFC cognitive functioning. NE improves working memory, response inhibition and lessens distractibility through actions at post-synaptic α2A adrenoceptors in the PFC, while DA improves working memory through modest stimulation of D1 receptors in PFC (reviewed in [20,21]). Although optimal levels of NE and DA are essential to proper PFC function, very high levels of NE and DA release, e.g. during stress, impair PFC function through α1, beta-1, D1 and possibly D4 receptors [22].

The current study characterized the effects of low, oral doses of MPH on PFC function in rats. Rats were tested on a working memory task, spatial delayed alternation, a classic test of PFC function in rodents [23]. MPH was found to have effects similar to those observed in patients: improving performance at moderate doses but producing perseverative errors at high doses. The second part of the study examined whether NE α2A adrenoceptor and/or DA D1 receptor actions contributed to the enhancing effects of MPH on PFC function.

## Results

### MPH dose/response: Effects on delayed alternation performance

**Figure 1:**
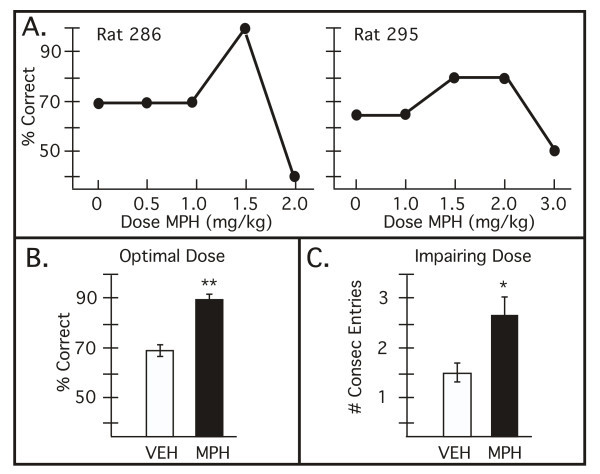
The effects of oral administration of methylphenidate (MPH) on delayed alternation performance in male rats. **A. **Representative dose/response curves from two individual rats. Results represent percent correct on the delayed alternation task following MPH administration. For most rats, a lower dose (1.0–2.0 mg/kg, p.o. 30 min) was found to improve performance, while higher doses often impaired performance (1.5–3.0 mg/kg). Rats showed individual differences in dose sensitivity. **B. **An optimal dose of MPH was found for all rats which significantly improved delayed alternation performance. Results represent mean ± S.E.M. percent correct on the delayed alternation task. VEH = cracker vehicle; MPH = optimal dose of methylphenidate (1.0–2.0 mg/kg); ** significantly different from VEH p = 0.002. **C. **Higher doses of MPH impaired performance and produced a perseverative pattern of errors. Perseveration was assessed by the greatest number of consecutive entries into a single arm of the T maze. Results represent mean ± S.E.M. number of consecutive entries. VEH = cracker vehicle; MPH = impairing dose of methylphenidate (1.5–3.0 mg/kg); * significantly different from VEH p = 0.046.

The effects of an acute, oral dose of MPH were examined over the dose range found to produce drug plasma levels in rats similar to clinical use in ADHD (0.5, 1.0, 1.5, 2.0 and 3.0 mg/kg, oral administration 30 min before testing). MPH produced an inverted U dose response curve whereby the middle doses (1.0, 1.5, 2.0 mg/kg) generally improved performance, while higher doses (2.0, 3.0 mg/kg) often impaired performance. Representative dose/response curves are shown in Figure 1A. There were individual differences in MPH dose sensitivity that may result from differences in MPH absorption from the gastrointestinal tract, and/or variations in endogenous catecholamine levels in PFC circuits. For all animals, a dose was found between 1.0–2.0 mg/kg that significantly improved delayed alternation performance (Figure 1B; vehicle vs. MPH p = 0.002, df = 7). No change in response time was noted (mean ± SEM vehicle: 191.2 ± 39.1 sec; mean ± SEM MPH: 179.3 ± 30.3 sec; p > 0.7, df = 7). These enhancing doses were used in subsequent experiments to examine the receptor actions contributing to MPH therapeutic actions.

Six of the eight rats tested showed impairment in delayed alternation performance as the dose was raised (1.5–3 mg/kg, especially following 2–3 mg/kg). The number of perseverative errors significantly increased at these higher doses, as measured by the greatest number of consecutive entries into a single arm (Figure 1C; p < 0.05, df = 5). A perseverative response pattern is consistent with PFC dysfunction. No consistent change in response time was observed following higher, impairing doses of MPH, although some animals were faster and some slower than usual (mean ± SEM vehicle: 201.4 ± 46.2 sec; mean ± SEM MPH: 194.2 ± 107.2 sec; range vehicle: 91–398 sec; range MPH: 50–675 sec). No stereotyped behaviors, common at much higher doses, were observed in these animals. Thus, cognitive choices, but not behavior per se, showed a perseverative profile.

### The role of α2 adrenoceptor mechanisms in the cognitive enhancing effects of MPH

**Figure 2:**
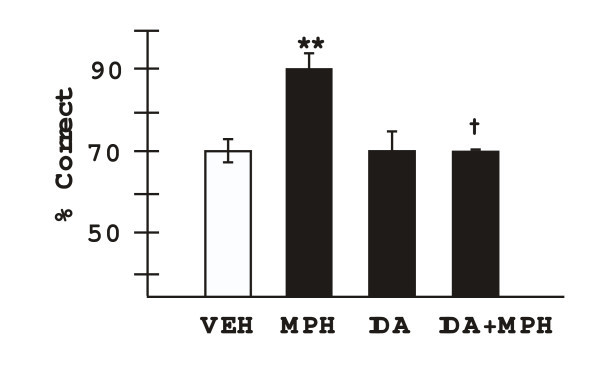
The enhancing effects of methylphenidate were blocked by co-administration of the α2 adrenoceptor antagonist, idazoxan at a dose which had no effect on its own. Results represent mean ± S.E.M. percent correct on the delayed alternation task. VEH = cracker vehicle; MPH = optimal dose of methylphenidate (1.0–2.0 mg/kg); IDA = idazoxan (0.1 mg/kg); ** significantly different from VEH, p = 0.002; † significantly different from MPH, p = 0.003.

The α2 adrenoceptor antagonist, idazoxan, was co-administered with MPH to test whether MPH enhances performance by facilitating endogenous NE stimulation of α2 adrenoceptors (n = 5). The optimal dose of MPH was selected for each animal; a dose of idazoxan was selected (0.1 mg/kg) that had no effects on its own. As shown in Figure 2, idazoxan significantly reversed the enhancing effects of MPH. Two way analysis of variance with repeated measures (2-ANOVA-R) showed a significant effect of MPH (F(1,4) = 18.45, p = 0.01), a trend toward significant effect of idazoxan (F(1,4) = 6.63, p = 0.06), and a significant interaction between the two drugs (F(1,4) = 27.2, p = 0.006). User defined contrasts showed that MPH+vehicle significantly improved performance compared to vehicle+vehicle (F(1,4) = 53.3, p = 0.002), while idazoxan+vehicle was not significantly different than vehicle+vehicle (F(1,4) = 0.02 p = 0.90). Most importantly, animals performed significantly lower on the delayed alternation task following MPH+idazoxan treatment than when they were administered MPH+vehicle (F(1,4) = 43.4, p = 0.0028), and were not significantly different in their performance from days in which they were administered vehicle+vehicle (F(1,4) = 0.005 p = 0.95). These results are consistent with α2 adrenoceptor actions contributing to the enhancing effects of oral MPH.

### The role of DA D1 receptor mechanisms in the cognitive enhancing effects of MPH

**Figure 3:**
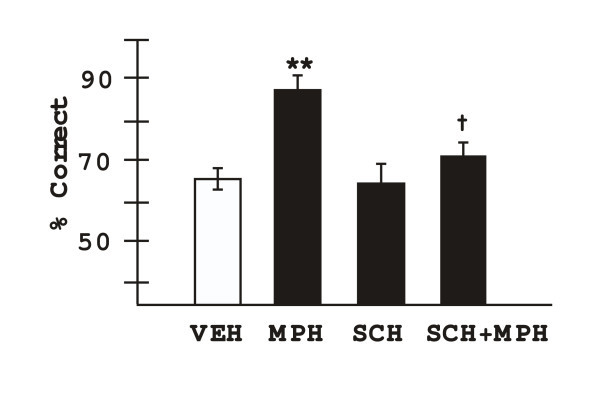
The enhancing effects of methylphenidate were blocked by co-administration of the dopamine D1 receptor antagonist, SCH23390 at doses which had no effect on their own. Results represent mean ± S.E.M. percent correct on the delayed alternation task. VEH = cracker vehicle; MPH = optimal dose of methylphenidate (1.0–3.0 mg/kg); SCH = SCH23390 (0.01 or 0.1 mg/kg); ** significantly different from VEH p = 0.0002; † significantly different from MPH, p = 0.008.

The DA D1 receptor antagonist, SCH23390, was co-administered with MPH to test whether MPH enhances performance by facilitating endogenous DA stimulation of D1 receptors (n = 7). A dose of 0.1 mg/kg SCH23390 was used, unless this dose produced impairment on its own. In these cases, the dose of SCH23390 was lowered to 0.01 mg/kg (n = 3). SCH23390 significantly reduced the enhancing effects of MPH on delayed alternation performance (Figure 3). 2-ANOVA-R showed a significant effect of MPH, a significant effect of SCH23390, and a significant interaction between the two drugs (effect of MPH: F(1,6) = 11.59, p = 0.014; effect of SCH23390: F(1,6) = 9.3, p = 0.023; interaction between MPH and SCH23390: F(1,6) = 9.3, p = 0.023). User defined contrasts showed that MPH+vehicle significantly improved performance compared to vehicle+vehicle (F(1,6) = 61.45, p = 0.0002), while SCH23390+vehicle was not significantly different than vehicle+vehicle (F(1,6) = 0.0, p = 1.0). Animals performed significantly worse on the delayed alternation task following MPH+SCH23390 treatment than when they were administered MPH+vehicle (F(1,6) = 15.0, p = 0.008). Although performance remained a bit above vehicle levels of response, performance following MPH+SCH23390 treatment was not significantly different than following vehicle+vehicle (F(1,6) = 0.84, p = 0.4). These results are consistent with D1 receptor actions contributing to the enhancing effects of oral MPH.

## Discussion

The current study provides the first evidence that oral dosing of therapeutically relevant levels of MPH improves PFC cognitive function in rats. This same dose regimen has been found to decrease locomotor activity in rats, reinforcing the idea that rats can be used as an appropriate animal model for examining medications used to treat ADHD patients.

Performance of the spatial delayed alternation task in a T maze is very relevant to many aspects of ADHD. Optimal performance of this task requires spatial working memory (remembering which side was most recently entered), response inhibition (inhibiting the tendency to return to the location where the animal was last rewarded) and the ability to sustain attention and suppress the distractions of being put into the start box. Thus, the delayed alternation task assesses many of the PFC operations that are known to be problematic in ADHD.

The current study found that moderate doses of MPH produced a highly significant improvement in delayed alternation performance. This improvement likely reflects enhanced PFC cognitive performance, as there were no changes in response time characteristic of motor or motivational changes. Indeed, given that MPH reduces eating, it is unlikely that simple changes in motivation for food reward could account for the improvement in performance.

It is also noteworthy that higher doses of MPH impaired delayed alternation performance in a large subset of animals. This impairment manifested in a perseverative profile of errors in which the rats continued to choose the same incorrect arm of the maze. Perseveration is also seen with 1) PFC lesions [23-26], 2) infusion of a high dose of a DA D1 or NE α1 agonist into the PFC [27,28], or 3) stress exposure, which causes high levels of NE and DA release in PFC [29]. Future studies will be needed to determine whether higher doses of MPH impair delayed alternation performance due to excessive catecholamine release in PFC, and if so, which receptor(s) underlie these impairing actions.

### Comparison to cognitive effects of MPH in humans

The MPH profile observed in rats in the current study is very similar to that in seen in humans. Oral administration of clinical doses of MPH have been found to improve spatial working memory, response inhibition, set-shifting and other PFC cognitive functions in both "normal" college students [30] and in children and adults with ADHD [31-35]. Imaging studies have shown more efficient dorsolateral PFC activity (BOLD) following MPH doses that improve spatial working memory, consistent with improved PFC cognitive function [36]. Interestingly, in adults with ADHD, childhood ratings of ADHD (both self-reported and informant ratings) correlated with response to methylphenidate on the spatial working memory task [35]. Thus, studies of spatial working memory performance are likely very relevant to the therapeutic effects of ADHD medications.

In the current study, higher doses of MPH impaired spatial working memory performance in a large number of animals. These findings are consistent with the original Lyon-Robbins analysis of stimulant actions in rodents that found increasingly perseverative responses with increasing dose of stimulant administration [37]. It is noteworthy that these "higher" doses are still much lower than those used in most other rodent studies, accentuating the fact that previous research in animals has often focused on MPH doses that are too high. Similar to our findings in rats, clinicians are often concerned that higher doses of MPH can induce perseverative thinking in patients e.g. [37,38]. For example, higher doses of MPH (e.g. 1.0 mg/kg) can increase perseverative errors on the Wisconsin Card Sorting Task when the test is novel [39,40]. Perseverative errors were not increased, and indeed were reduced by MPH, when the Wisconsin Card Sorting Task was given repeatedly [41]; however, this condition minimizes the need for flexible thinking, as the switching rule is "discovered" only during the first experience of the task. Douglas et al. also found no evidence of perseveration on other tasks, such as the Trails B, and concluded that doses below 0.9 mg/kg produced dose-related improvements in cognitive flexibility. As repeated daily doses can add together, they cautioned that doses above 0.6 mg/kg were not recommended. Thus, under optimal dosage conditions, MPH appears to improve flexible thinking in patients, but higher doses may produce a perseverative profile similar to that seen in rodents.

### Receptor mechanisms underlying PFC cognitive enhancing effects of MPH

The identification of an MPH dose regimen that improves PFC cognitive performance in rats provides the opportunity to examine the neural mechanisms underlying MPH therapeutic actions. The current study began by examining the role of NE α2 and DA D1 receptors, given the importance of these receptors to PFC cognitive function. The study found that both the α2 antagonist, idazoxan, and the D1 antagonist, SCH23390, reversed the cognitive-enhancing effects of MPH. Care was taken to use antagonist doses that did not impair performance on their own; thus, additive effects of drug treatment cannot account for the normalization of response. Rather, the data are consistent with MPH improving performance by increasing the availability of NE and DA, which in turn stimulate α2 and D1 receptors. It is interesting that either idazoxan or SCH23390 was fully effective in reversing the MPH response. These data suggest that there may be beneficial interactions between these receptors, an area that has received little investigation.

Future studies will be needed to determine whether the enhancing effects of MPH occur in the PFC and/or elsewhere in the brain. Low, systemic doses of MPH and amphetamine are known to increase both NE and DA levels in the rat PFC while having more subtle effects in striatal regions ([19], and C.W. Berridge, personal communication). There are relatively low levels of DA transporters in the PFC [42]; thus the increase in both DA and NE levels likely occurs through blockade of NE transporters, which are thought to transport both NE and DA in the PFC [42].

The efficacy of idazoxan and SCH23390 in reversing the MPH response in the current study is not unexpected, given the importance of α2 and D1 receptor actions to PFC cognitive function. Catecholamine depletion in PFC produces working memory deficits as severe as ablation of the tissue itself [43], and D1 receptor blockade similarly weakens working memory regulation of behavior [44,45]. In the current study, D1 blockade did not appear as completely effective as α2 receptor blockade, although both antagonists weakened the improvement such that it was statistically insignificant from vehicle. The potentially weaker effects with SCH23390 may be due to the lower dose used in some animals, and the difficulties in dealing with an inverted U dose response, where either too little or too much D1 receptor stimulation can impair performance. Under these conditions it is difficult to identify the correct dose of antagonist to perfectly normalize behavior. Individual variability in response to SCH23390 may arise from differences in endogenous D1 receptor stimulation under basal conditions. For example, D1 agonists have been shown to enhance attentional control in rats when infused into the PFC, but only in animals that were performing relatively poorly under basal conditions [46]. D1 receptors also play a key role in striatal function, and it is possible that these actions outside the PFC also contributed to the enhancing effects of MPH on the delayed alternation task.

With some notable exceptions [47,48], much of the ADHD field has focused on DA mechanisms in ADHD [49]. In turn, there has been intensive focus on the DA actions of MPH, with some researchers even referring to MPH as a selective DA transporter blocker. The current data, in addition to recent biochemical studies [11,18,50] caution that the NE actions of stimulants such as MPH are just as important as the DA effects. This point is accentuated by the findings that one can recreate the symptoms of ADHD- increased locomotor activity, poor impulse control and weakened working memory/distractibility- by blocking α2 adrenoceptors with yohimbine infusions in the monkey PFC [51-53], respectively. Yohimbine also reduces the delay-related activity of PFC neurons, the cellular measure of working memory and response inhibition [54]. Conversely, the α2 agonist, guanfacine, has been shown to strengthen working memory [55,56], reduce distractibility [57], improve response inhibition [58-60], and increase regional cerebral blood flow in monkey PFC [61]. Most recently, guanfacine has been shown to reduce locomotor hyperactivity and improve attentional control in the spontaneously hypertensive rat, a rodent model of ADHD (T. Sagvolden, personal communication). Experiments are in progress to determine whether MPH loses efficacy in mice with a functional knockout of the α2A adrenoceptor. The current results with idazoxan indicate that at least some of the beneficial effects of MPH arise from NE stimulation of α2 adrenoceptors.

### Relevance to medications used to treat ADHD

Many of the medications used to treat ADHD increase endogenous NE stimulation of α2 adrenoceptors or mimic NE by directly stimulating these receptors. For example, like MPH, atomoxetine (Strattera) and amphetamine (Adderall) increase NE as well as DA in the PFC of rats [19,42]. NE reuptake blockers have been shown to be very efficacious in treating ADHD symptoms, although their cardiac side effects have limited clinical utility in children [47,62]. Future studies will need to examine whether low, oral doses of amphetamine and atomoxetine, like MPH, can improve delayed alternation performance in rats. Guanfacine mimics NE at α2 adrenoceptors, and is now in common use for the treatment of ADHD, especially in patients with tic disorders or drug abuse liability who cannot take stimulant medications [63]. Guanfacine has been shown to improve spatial working memory performance in mice [64], rats [65], monkeys [55,66] and humans [67]. Thus, there is an excellent correspondance between drug effects in the laboratory and clinical efficacy in ADHD. The identification of an appropriate MPH dose regimen for use in rats should help in the development of safer and more effective medications for the treatment of ADHD.

## Conclusion

Low, orally-administered doses of MPH improve spatial working memory performance in rats, while higher doses often impair performance and induce perseverative errors. Both NE α2 adrenoceptor and DA D1 receptor stimulation contribute to the enhancing effects of MPH on working memory in rodents. Future studies with low, oral doses of MPH may continue to elucidate the mechanisms underlying the therapeutic actions of MPH in treating ADHD.

## Methods

### Animals

Young adult (240–260 g) male rats were purchased from Taconic (Germantown, NY) and singly-housed in filter frame cages. Animals were kept on a 12 hr light/dark cycle, and experiments were conducted during the light phase. Rats were slowly habituated to a restricted diet (16 gm/day per rat) of autoclaved Purina (St. Louis, MO) rat chow during the first two weeks. Food was given immediately after behavioral testing and water was available *ad libitum*. Rats were weighed weekly to confirm normal weight gain. Food rewards during cognitive testing were highly palatable miniature chocolate chips. Rats were assigned a single experimenter who handled them extensively before behavioral testing.

### Cognitive assessment

Rats were habituated to a T-maze (dimensions, 90 × 65 cm) until they were readily eating chocolate chips placed at the end of each arm and were acclimated to handling. After habituation, rats were trained on the delayed alternation task. On the first trial, animals were rewarded for entering either arm. Thereafter, for a total of 10 trials per session, rats were rewarded only if they entered the maze arm that was not previously chosen. Between trials the choice point was wiped with alcohol to remove any olfactory clues. The delay between trials started at "0" sec (i.e. about 1.5 sec, minimum possible for delayed alternation) and was subsequently raised in 5 sec intervals as needed to maintain performance at about 70% correct. Animals were scored for accuracy (arm chosen) and response time for each trial.

### Drug administration

The experimenter testing the animal was unaware of drug treatment conditions. Given the need for oral administration of drug, rats were habituated to eating a small piece of cracker. All animals had learned to ingest the cracker rapidly and completely prior to the initiation of drug testing.

MPH was acquired from the National Institute of Drug Abuse. Doses were based on those identified by Kuzcenski and Segal [11]. MPH was dissolved in tap water and injected onto a small piece of Saltine cracker that was fed to the rat 30 min before cognitive testing. The doses examined were: 0 (water only), 0.5, 1.0, 1.5, 2.0, and 3.0 mg/kg). For example, the 1.0 mg/kg dose was made by dissolving 1 mg MPH in1 ml water and injecting a volume equivalent to the weight of the rat, e.g. a 450 g rat would receive 0.45 ml injected onto the cracker. Animals rapidly ate the cracker once habituated to the procedure. Doses were administered in random order with the exception that no animal began with the 3.0 mg/kg dosage.

Idazoxan was purchased from Sigma (St. Louis, MO) and administereed at a dose of 0.1 mg/kg. Idazoxan was dissolved in saline, and like MPH, was injected into the cracker for oral administration.

SCH23390 also was purchased from Sigma and dissolved in saline. SCH23390 was initially administered in a dose of 0.1 mg/kg. Animals who were impaired by this dose were subsequently administered 0.01 mg/kg SCH23390 so that additive effects could not account for MPH reversal.

### Data analysis

The dependent variables were percent correct on the delayed alternation task (accuracy), response time, and greatest number of consecutive entries into an incorrect arm (perseveration score). Statistical comparisons utilized within subjects designs; simple comparisons utilized paired (dependent) T tests. The effects of idazoxan or SCH23390 on the MPH response were analyzed with a two-way analysis of variance with repeated measures with factors of 1) MPH and 2) antagonist, and user defined contrasts to test pairwise comparisons.

## Authors' contributions

A. Arnsten designed the study, analyzed data, drew the figures and wrote the paper. A. Dudley, in collaboration with the laboratory technicians, made up drug, tested the animals on the spatial working memory task, and helped with data analysis and the writing of this paper.
